# Human Initiated Cascading Failures in Societal Infrastructures

**DOI:** 10.1371/journal.pone.0045406

**Published:** 2012-10-31

**Authors:** Chris Barrett, Karthik Channakeshava, Fei Huang, Junwhan Kim, Achla Marathe, Madhav V. Marathe, Guanhong Pei, Sudip Saha, Balaaji S. P. Subbiah, Anil Kumar S. Vullikanti

**Affiliations:** 1 Network Dynamics and Simulation Science Laboratory, Virginia Bioinformatics Institute, Virginia Tech, Blacksburg, Virginia, United States of America; 2 Department of Computer Science, Virginia Tech, Blacksburg, Virginia, United States of America; 3 Ericsson, San Jose, California, United States of America; 4 Microsoft Corporation, Seattle, Washington, United States of America; 5 Department of Electrical and Computer Engineering, Virginia Tech, Blacksburg, Virginia, United States of America; 6 Department of Agricultural and Applied Economics, Virginia Tech, Blacksburg, Virginia, United States of America; 7 NetApp, Inc., Research Triangle Park, North Carolina, United States of America; Northeastern University, United States of America

## Abstract

In this paper, we conduct a systematic study of human-initiated cascading failures in three critical inter-dependent societal infrastructures due to behavioral adaptations in response to a crisis. We focus on three closely coupled socio-technical networks here: (i) cellular and mesh networks, (ii) transportation networks and (iii) mobile call networks. In crises, changes in individual behaviors lead to altered travel, activity and calling patterns, which influence the transport network and the loads on wireless networks. The interaction between these systems and their co-evolution poses significant technical challenges for representing and reasoning about these systems. In contrast to system dynamics models for studying these interacting infrastructures, we develop interaction-based models in which individuals and infrastructure elements are represented in detail and are placed in a common geographic coordinate system. Using the detailed representation, we study the impact of a chemical plume that has been released in a densely populated urban region. Authorities order evacuation of the affected area, and this leads to individual behavioral adaptation wherein individuals drop their scheduled activities and drive to home or pre-specified evacuation shelters as appropriate. They also revise their calling behavior to communicate and coordinate among family members. These two behavioral adaptations cause flash-congestion in the urban transport network and the wireless network. The problem is exacerbated with a few, already occurring, road closures. We analyze how extended periods of unanticipated road congestion can result in failure of infrastructures, starting with the servicing base stations in the congested area. A sensitivity analysis on the compliance rate of evacuees shows non-intuitive effect on the spatial distribution of people and on the loading of the base stations. For example, an evacuation compliance rate of 70% results in higher number of overloaded base stations than the evacuation compliance rate of 90%.

## Introduction

The social and economic well being of a modern society depends on the reliable functioning of the critical infrastructures such as energy, transportation, communications, banking, and water system. Historically, these critical infrastructures have been physically and logically disconnected and had very little interdependence. However, advances in cyber-based systems and communication infrastructure have coupled these systems. This coupling, on one hand has made many of the systems more robust and efficient, but on the other hand, the close coupling has introduced new vulnerabilities in these inter-dependent systems. Failure in one system can cascade and cause failures in other coupled systems. We call such cascades *co-evolving cascades in inter-dependent infrastructures*. See [1]–[7] for further discussion on the duality between fragility and robustness of infrastructure systems. Most of the research in the literature has concentrated on cascades between infrastructures that result from their physical connectivity. Recent crises including hurricanes, the north east blackout and the 9/11 incident [8] are examples where failures propagate across infrastructures due to loss of communication and electricity.

However, another source of interdependency arises from *human initiated cascades*, e.g., behavioral changes in demand (this is discussed in greater detail in Section *Human Initiated Cascades*). For instance, wireless cellular network traffic is greatly influenced by the activities of the population. Natural and human-initiated disasters can cause significant changes in activities and road traffic (e.g., as a result of an evacuation from the affected area), which can, in turn, significantly alter the calling pattern and the characteristics of the wireless cellular network traffic. This is fundamentally different than physical inter-dependencies and is the focus of the present work.

### Human Initiated Cascades

**Figure 1:**
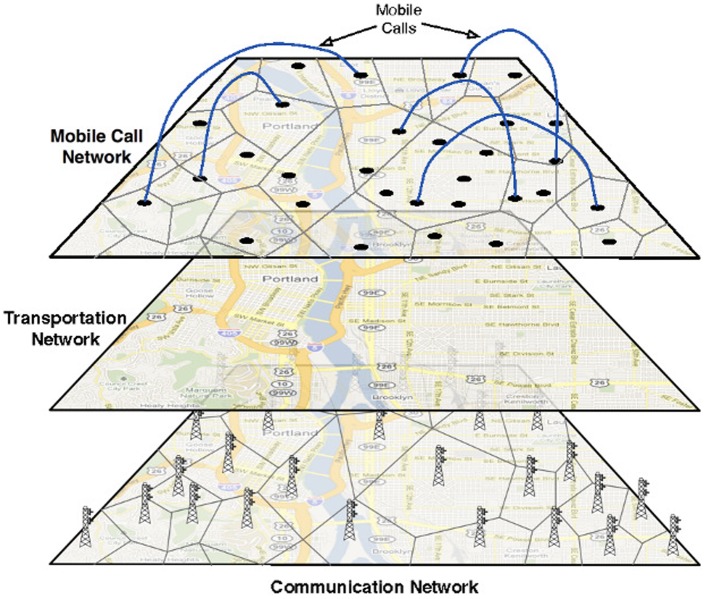
Layered architecture of the inter-dependent infrastructures. The bottom layer shows the communication network, the middle layer represents the transportation network and the top layer shows the call graph at a particular instant.

We develop a conceptual framework and associated modeling environment for studying *human initiated inter-dependencies between critical societal infrastructures* and their role in initiating, and responding to cascading failures of such inter-dependent infrastructure systems. Specifically, we study the interdependencies between the transportation and communication infrastructures and study methods to identify critical base stations and regions, quantify the impact on the overall traffic (e.g., in terms of number of calls served/dropped), and techniques to mitigate the effects. Figure 1 shows the layered architecture of the inter-dependent infrastructures.

The role of humans in such situations is at least two-fold: (i) as consumers using functioning infrastructures to fulfill their demands; and (ii) as operators, managers and policy makers who are responsible for maintaining, upgrading and operating the infrastructures. Both of these interactions produce human-mediated interdependencies. Two examples clearly demonstrate both facets (consumer and operator) of interaction. The demand for cooling on a hot summer day can strain the energy distribution system, forcing it to operate in a less robust regime. Furthermore, the consequences of decisions made to mitigate accidents depend on the demand being serviced at the moment. Thus a decision to brown-out New York's financial district while maintaining service to residential areas has completely different effects at midnight on a Saturday than at 2:00PM on a Wednesday.

These human-initiated cascades and interactions can have consequences just as important as the physical interactions, and this paper focuses on the former kind of interdependency. This is especially true in the context of a large-scale crisis where people's reactions may produce very unusual patterns of demand. It is impossible to measure how the demand on various infrastructures changes when components fail – not only would experiments on the real system be unethical but the systems also evolve too rapidly and the number of possible failures is too large to study from observations of accidents. In a recent report Peeta et al. [9] point the need for developing individualized modeling approach that takes into account the perception of risk and the consequent behavior in the event of an evacuation. Our earlier results [10] also point to a similar need. From a theoretical standpoint, the problem actually becomes one of control when the system is constantly adapting rather than prediction, and as such computational results show that developing closed form analytical solutions is unlikely [11]. Hence, estimating total demand, its geographic distribution, and how it is affected by changes in infrastructure is best accomplished by understanding the behaviors that lead to demand for services – in other words we need a first principles individualized modeling approach.

As a simple example, we can combine estimates of calling patterns of all individuals based on their demographics and location to generate a spatio-temporal estimate of aggregate call load on base stations. The required estimates can be calibrated and validated against demand data from typical days as well as entirely different kinds of data, such as traffic counts, land use, and time use surveys. Although the data from typical days offers no insight into abnormal days, by capturing the behavioral causes of observed aggregate demand at the individual level, we can indeed generalize to hypothetical situations. Thus, in the event of a biological or chemical attack, individuals and local authorities might seek evacuation of the affected area. The exact time varying loads on the base stations due to the new calling patterns (resulting from additional calls individuals are likely to make for coordination and the traffic congestion) cannot be understood without representing the behavioral component.

### Overview of the Scenario

Understanding the complexity and interdependencies among critical infrastructures requires detailed representations of these systems as well as points of interactions. The precise impact on the cellular network infrastructure depends crucially on the models and parameters (e.g., of behavioral changes in calling patterns), and simple aggregate models for survivability analysis are often inadequate [4], [9], [11]. In this paper, we use a realistic synthetic network model with end-to-end representation of detailed models of transportation, and communication systems [12]–[15], for the region of Portland, Oregon (see Section *Formal Models and Methodology* for more details on these models). We consider a simple *no-notice* event that is based on Homeland Security planning scenarios. The evacuation in this scenario is not predicted and no partial evacuation has taken place. Furthermore, since the event can happen anywhere in the city, planning is necessarily done at a fairly high level. Table 1 provides a summary of the figures that describe the scenario. Table 2 provides a list of figures that display the variation in the spatio-temporal load and Table 3 lists a summary of all the tables.

**Table 1:** Summary of figures for experiment setting.

Figure	Comment
Figure 1	Layered architecture of the inter-dependent infrastructures
Figure 2	Evacuation region we consider for the study
Figure 3	Time line of the evacuation event
Figure 4	Cascades in inter-dependent networks A: Causal diagram B: Artwork
Figure 5	Input and output statistics for the SG call patterns
Figure 8	Locations of Burnside Bridge and Morrison Bridge which are closed in Evacuation Scenarios

**Table 2:** Summary of figures for spatio-temporal load.

Figure	Quantity	Sub-figure (a)	Sub-figure (b)	Time Interval	Comment
Figure 9	Deficit capacity	Before cascading	After cascading	1:00PM–1:30PM	Evac I-a
Figure 12	Load	Evac I-a	Evac I-b	12:00PM–12:30PM	90% Compliance
Figure 13	Load	Evac I-a	Evac I-b	12:00PM–12:30PM	70% Compliance
Figure 14	Load	Evac I-a (70%)	Evac I-b (90%)	12:00PM–12:30PM	70%, 90% Compliance
Figure 15	Load	Evac I-a (70%)	Evac I-b (80%)	12:00PM–12:30PM	70%, 80% Compliance
Figure 16	People Intensity	Base case	Evac I-a	Entire day	
Figure 17	Scenarios	Total calls	Mobility	Entire day	
Figure 18	Scenarios	Evacuation Region	Shelter Area	Entire day	
Figure 19	Load	Base case	Evac I-a	1:00PM–1:30PM	
Figure 20	Load	Evac I-a	Evac I-b	1:00PM–1:30PM	
Figure 21	Load	Evac I-a	Evac I-b	1:00PM–1:30PM	Zoom-in of Figure 20
Figure 22	Load	Evac II-a	Evac II-b	1:00PM–1:30PM	

**Table 3:** Summary of tables.

Table	Comment
Table 1	Summary of Figures for Experiment Setting
Table 2	Summary of Figures for Spatio-temporal Load
Table 3	Summary of Tables
Table 4	Some basic statistics about the evacuation
Table 5	Simulation Study Cases
Table 6	Congestion Analysis for Time 12:00PM–12:30PM with Call Capacity Threshold = 250
Table 7	Congestion Analysis for Time 12:00PM–12:30PM with Call Capacity Threshold = 750

**Figure 2:**
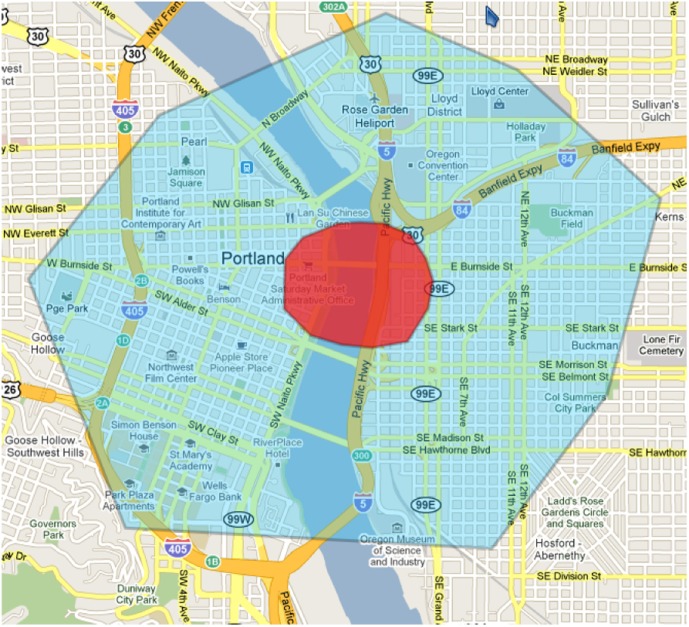
Evacuation region we consider for the study. The harmful chemical agent is released in the polygonal area colored in red at the center. The region around, colored in blue is the evacuation region.

**Figure 3:**
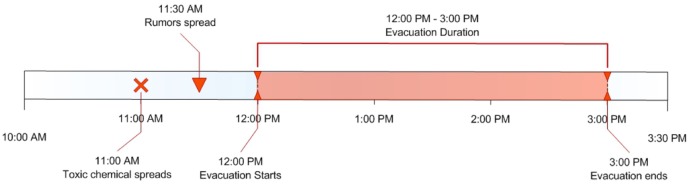
Time line of the evacuation event. **11:00AM** A chemical agent is released at a place near downtown Portland; **11:30AM** Rumors spread about an impending evacuation; **12:00PM** Evacuations begins; **3:00PM** Chemical pollution is cleared and the evacuation ends.

A chemical agent is assumed to be released in a spot near downtown Portland at 11:00AM, when the downtown area is full of office workers, shoppers and commuters. The toxic chemical spreads in vaporized form forming a plume. Figure 2 shows the plume and the evacuation area and figure 3 illustrates the time line of this scenario. It is determined (by the planning authorities) that the spread of the toxic chemical requires everyone within several blocks of the release and plume area (about 174,536 individuals) to be moved to the outskirts of the city, starting at noon. The red region is the plume area and the blue region is the affected area. Hence people need to be evacuated from both the red and the blue regions. People whose home is outside the evacuation area are sent home, while others are sent to a designated evacuation shelter. For residents outside the evacuation area, it is assumed that “in-place” shelter for about a day affords the best protection. Though the evacuation is ordered around noon, we assume that people have self-warning signs (e.g., smell or other physiological symptoms), and start asking for and spreading information about the release from 11:30AM (e.g., rumors about an impending evacuation), and start making more calls to their family and friends. Starting at noon, the evacuation begins. By 3:00PM, the chemical pollution is cleared up by the authorities, and the evacuation is announced off.

**Figure 4:**
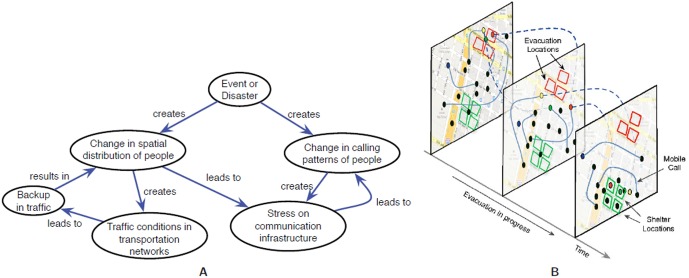
Cascades in inter-dependent networks. **A:** Causal diagram that depicts the role of human initiated cascades in our scenario. The cascade is caused due to two interacting components: (i) individuals change their calling patterns and (ii) evacuation causes modifications to people's traveling pattern and hence loads on the base stations. Evacuation leads to traffic jams and thus a simple statistical predictive model of calling patterns is not adequate. In our current scenario, individuals do not change their driving routes dynamically. **B:** Artwork describing how the evacuation like scenario plays out in space and time. Both evacuation areas (red squares) and temporary shelters (green squares) are marked. The dotted lines joining a red dot (individual) depict the individual's movement as he proceeds to the shelter. The solid lines denote calls. Note the change in the spatio-temporal calling network as a result of the movement.

During the evacuation, the roads leading out of this region get congested, and people make additional mobile calls; see Section *Evacuation Model* for more details on the scenario. Figure 4 depicts the role of human initiated cascades through a causal diagram as people's travel pattern and calling behavior change. We use simple parameterized models to study the modified calling behavior, and study its impact on base station loads. As discussed later, several base stations around the affected region get overloaded, resulting in a significant fraction of call drops. Note that this cascade in the breakdown of infrastructures is human mediated since it is caused by the modified individual and component level behavior.

Many approaches have been developed for dealing with the issue of overloaded base stations. One common strategy is to allow heterogeneous overlapping coverage regions [16] – this can be accomplished by means of directional antennas with variable power levels. However, there are limitations to this approach, since arbitrary and high overlap can impact the channel efficiency. Another strategy that has been studied is to augment the cellular network with wireless ad-hoc/mesh connectivity, possibly at the expense of increased delays, e.g., Law et al. [17]. The energy usage and the delays resulting from it (which are proportional to the maximum number of hops) are important metrics in such an approach. We study simple techniques to augment capacity using this approach.

The scenario is *idealized* and does not provide details on the chemical plume, including, method of delivery, its shape and its evolution, its progression in time, detection and physiological effects. Further, we assume a simplified model of mobility during the evacuation, and parameterize various kinds of behavioral changes, such as in calling patterns, since it is difficult to obtain real measurements for them. All the assumptions used in this study are discussed in detail in Section *List of Assumptions* in Appendix S1. Nevertheless, it is sufficiently rich to motivate the need for understanding three important issues that are the focus of this paper: (i) individual and system level behavioral adaptations and their role in cascading failures, (ii) couplings between the infrastructures and methods for representing and reasoning about them, and (iii) individual-based representation of the infrastructures and the social system.

### Summary of Contributions

We develop an integrated modeling environment to study *human initiated* cascading failures in coupled transportation, social and cellular infrastructure systems. Many of the ideas are generic and can be applied to study other coupled infrastructures as well. The broad contributions in our paper are: (i) we develop an individual based modeling framework for representing the social, transportation and cellular systems [12], [14], [15] – our approach is based on recent results that show that such individual-based approaches often provide very different qualitative insights than the traditional system dynamics models [18], [19](In case of epidemiological models, there have been instances where the opposite has been shown to be true, e.g. see [20]), (ii) we conduct an illustrative case study that demonstrates this modeling environment – the study is chosen to highlight the individual behavioral adaptation in the event of a no-notice crisis and its emergent effect on multiple infrastructures and the feedback that results from these interactions. The modeling environment provides policy makers and analysts a way to compare various response strategies and ‘what-if’ scenarios. Our specific contributions include:

1. *Construction of a first principles based approach for synthetic wireless cellular network traffic during an evacuation*: Building on our approach from [12]–[15], we develop a model for wireless cellular network traffic during an evacuation in the downtown region of Portland, OR. This involves the following technical extensions: (i) construction of an instance of the activity based mobility model based on [12]–[15], with changes in activity patterns during the evacuation, and (ii) modifications in the calling patterns for individuals during the evacuation period, based on their location and activity. In this study, we use simple parameterized models for the two behavioral adaptations; the system itself can handle more complicated behavioral adaptation. Note that the two behavioral adaptations are coupled in that individuals use their devices while evacuation is underway. This induces time-varying loads on both these infrastructures; furthermore, the time series of the loads cannot be decoupled due to feedbacks.
2. *Analysis of altered traffic characteristics during evacuation*: We compare the traffic characteristics during evacuation time with a baseline involving normal activities, and find a significant difference in the base station loads around the evacuation region, as well as around the shelter; the differences depend crucially on the calling behavior. The load characteristics show high variation at different base stations, depending upon their location. We also check if there are significant call drops around the evacuation region, in order to identify critical cell towers.
3. *Sensitivity of geographic distribution of people and the base station load to the evacuation compliance rate*: Contrary to our expectation, the results show that higher evacuation compliance rate and hence a larger number of evacuees result in fewer base stations to be overloaded. This is because as more people try to flee the plume area, the traffic congestion results in higher concentration of their geographic location, which further makes the base station loads to be concentrated to a few base stations. Similarly road closures show interesting emergent phenomenon as explained in detail under Section *Sensitivity Analysis of Evacuation Compliance*.
4. *Methods to design hybrid networks to improve capacity*: We study a simple iterative strategy to balance the load in a hybrid mesh and cellular network, in which the excess load on a base station is routed to its neighboring cells (by means of the multi-hop mesh network, which is assumed to be added for dealing with the evacuation. We find that the number of hops allowed (which corresponds to the extra delays for some of the calls) has a significant impact on the call drops.

### Related Work

Recently there has been substantial interest in studying cascading failures, and methods to prevent them, in interdependent critical infrastructures; see [3]–[6], [10], [11], [21]–[27] and a detailed but slightly older survey [4]. Reliability and robustness of individual societal infrastructures have been actively studied within their respective fields for quite some time now; see [3], [5], [6], [11] and the numerous references therein. Theoretical models and analytical results for studying issues related to vulnerability and criticality can be found in several papers including [1], [11], [28], [29]. Interdependency in infrastructural networks has also been analyzed numerically and analytically where a subset of nodes from each network depend on other networks [30]–[32].

There are essentially three progressively more realistic (and usually computationally more expensive) methods for assessing reliability – (i) static methods that view the system as a graph or fixed object and look for ways to *break or shatter* the graph by removing components of it, (ii) a steady state analysis that usually takes form of looking for flows in these graphs and analyzing the effects of removing one or several components on the resulting flow and (iii) a dynamical analysis which looks for faults and disturbances that are cascading and are inherently dynamic in nature. We focus on the third method here and use an interaction based modeling framework [21], [22] – the modeling framework although similar to the well known agent-based modeling framework differs in substantial ways.

Work by [33], [34] calls for behavioral and decision constraints to be taken into account when planning and managing evacuations and disasters. Authors in [35], [36] identify social and environmental factors that help in predicting the evacuation behavior of the individuals. Behavior adaptation in other socially disruptive situations have been studied by [37], [38].

Expertise in multiple networked infrastructures can help in a trans-disciplinary approach to study critical infrastructures. For example, to study the effect of mobile calling patterns under varying traffic scenarios on the cellular infrastructure, expertise in mobility models and social and behavioral modeling of calling patterns are required. Modeling wireless cellular network traffic under the scenario of traffic evacuation is trans-disciplinary research which requires expertise in multiple networked infrastructures, social and behavioral modeling, mobility modeling, cellular and wireline sessions generations etc. Existing research focuses on individual infrastructures rather than their interdependencies. For instance, work by [12], [39] focus only on the wireless and communication networks, while [40]–[42] model vehicular traffic in case of evacuation. The focus of this research is to study and analyze the interaction between the communication and transportation infrastructures as people change their calling patterns while stranded in the traffic congestion or during disasters. It incorporates detailed models of individual behavior as people deviate from their usual activity patterns to adjust to the environment around them.

Our previous work [12] models the Primary User (PU) behavior in cellular networks, synthesizes and analyzes the spatio-temporal spectrum demand patterns. We developed a methodology to generate synthetic network traffic data to model primary usage, by combining a number of different data sets for mobility, device ownership and call generation in a large synthetic urban population. Unlike simple random graph techniques, these methods use real world data sources and combine them with behavioral and social theories to synthesize spatial and dynamic relational networks [43]. In this paper, we extend the prior work to study the impact of vehicular traffic congestion resulting from an evacuation scenario on the wireless spectrum usage in the region [44].

Traffic modeling in terms of evacuation has been extensively studied in previous works, such as [40]–[42], [45], [46]. [40] models the evolution of multi-commodity traffic flows over complex networks during evacuation, based on a simple macroscopic computer representation of traffic flow that is consistent with the kinematic wave theory under all traffic conditions. [41] presents a model reference adaptive control (MRAC) framework for real-time traffic management under emergency evacuation. Location allocation model that reduce highway clearance time by selecting from candidate shelters is studied in [45]. An emergency hurricane evacuation planning module is proposed in [46]. An analytical solution for the critical fraction of nodes that, on removal, lead to a concurrent failure and a complete fragmentation of two interdependent power and control networks is provided in [47]. However, previous work in traffic modeling has no discussion about how the traffic congestion resulting from evacuation impacts the wireless spectrum usage in the region. A few studies in the past, have tried to identify traffic congestion areas by observing base station dwell time–the length of time a mobile device stays registered to a base station in a cellular network. However, our study looks at the inverse of this problem. It analyzes not only the interplay between the infrastructures but also identifies the criticality of base stations that are responsible for cascading failures of other base stations.

### Formal Models and Methodology

We represent the inter-dependent critical infrastructures and the society they serve as *layered coupled and co-evolving graphical dynamical systems*; see [11] for formal definitions. Informally, such a system has a set of networks, the vertices and edges in each network represent the individual entities and their direct interactions (dependencies). In the present paper, we have four distinct interacting networks (partly illustrated in Figure 1).

1. a hybrid wireless network with its constituent elements (base stations, switching centers, radio and wired links, etc.)
2. a transportation network with roadway links and specific physical locations, and
3. a mobile call network, in which vertices represent individuals residing in an urban region, with edges between individuals who participate in a call.
4. A social network where vertices represent individuals and edges represent relationships, such as co-location or friendship.

These layers are also connected; in our case this connectivity is primarily obtained via the spatial social network. The calls constitute an appropriate load on the wireless network. As individuals are evacuated, they use the underlying transportation network and potentially pre-specified set of routes. They also change their calling patterns. Together this yields new load patterns on the wireless network. Thus the inter-layer edges capture the load on both the infrastructures – this network connectivity is often not physical.

### Representation of the Event

A crisis event among other things consists of the following components: (i) intensity, (ii) spatio-temporal patterns, (iii) predictability or estimated time of the event and (iv) level of destruction of the physical components. In our scenario, we consider a biological or chemical agent that is released either deliberately or accidentally. This is what has been termed as *no-notice* or *flash event*. We assume that every individual under the plume can be affected. In this paper, we do not consider the health impact of the release; this topic is subject of another paper. We also assume that the plume is represented as a convex polygon. This is a very coarse representation when dealing with health impacts but one that suffices to understand the loads on transportation and wireless networks. Other geometrically decaying, spatially oriented representations can be considered if needed.

### Individuals, Behaviors and Risk

A key component of our paper is to represent individual behaviors in the event of a crisis. At a minimum, individual behavior depends on the person's demographics (gender, age, income, risk-tolerance, etc.) as well as the perception of risk. Risk perception further depends on the available information about the crisis (where has it happened, how dangerous it is, how quickly is it going to spread, etc.). In this paper, we assume that due to differences in risk perceptions, attributes and behavior, everyone in the evacuation area does not want to comply with the evacuation orders. In fact only 90% of the affected people actually evacuate. We further assume that individuals have been assigned specific shelters to go to and they will take shortest perceived paths—the paths of-course depend on the loads on the roadway and this is accounted for in our routing algorithm.

### SSRSM: A High Level Description

This paper uses our tool *Synthetic Spatio-temporal Relational Session Modeling Environment* (SSRSM) for synthetic wireless cellular network demand modeling; see [12] and Section *Related Work* for details. SSRSM constructs an activity based model for urban mobility, and then produces a model for network traffic. In this paper, we modify the activity sequences to model evacuation from downtown Portland, OR, to obtain an altered mobility model. This is then combined with calling patterns that reflect the changes due to evacuation (e.g., people being evacuated would make more frequent shorter calls). Our paper builds on SSRSM, but additional changes are needed to incorporate the evacuation scenario. We discuss below a high level description of SSRSM and then the modifications needed to model the evacuation. SSRSM consists of three modules: (i) realistic activity based mobility model, (ii) device ownership model, and (iii) wireless session generation model; these are briefly discussed below.

#### Activity Based Mobility Model

The basic mobility patterns were constructed using the methodology from [15], [48], [49] for modeling activity based synthetic populations and urban mobility; this methodology involves a multi-step process, which starts with the creation of synthetic urban populations by integrating a variety of databases from commercial and public sources into a common architecture for data exchange that preserves the confidentiality of the original data sets, and yet produces realistic attributes and demographics for the synthetic individuals. It further determines a set of activity templates for individuals in the households, based on US census and survey data on activity and time-use surveys. These activity templates describe the sort of activities each household member performs and the time of day they are performed. Statistical models from transportation literature are used to choose activity locations for all individuals, who are then routed on shortest paths between these locations. The output of this component is a minute-by-minute schedule of each person's activities and the locations where these activities take place. The output also yields a co-location based dynamic social contact network, which updates the social contacts for each person on-the-fly according to his/her activities and their respective locations. Most of our analysis of the calling pattern metrics is done at a base station level. We divide the Portland region into uniform sized cells, and use this mobility model to track the sequence of cells traversed by each person.

#### Wireless Device Ownership

We use data from the 2007 National Health Interview Survey (NHIS) of about 29,000 households from the National Center for Health Statistics of the Center for Disease Control [50]. The survey data includes demographic information such as household size, household income, householder's age, number of workers in a household, number of cells phones in a household, and has a device penetration of 53.44%. SSRSM uses the well known techniques of classification and regression trees (CART) [51], adapted for synthetic populations [52] and constructs regression trees based on the demographic information that best describe the allocation of cell phones among householders.

#### Modeling Calls and Usage Patterns

**Figure 5:**
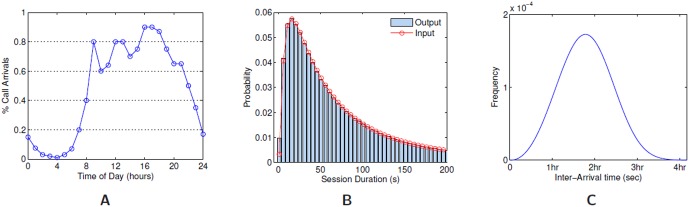
Input and output statistics for the SG call patterns. **A:** The number of mobile call arrivals at different times of day are taken from the data reported in [55]. **B:** Mobile and landline session durations follow a LogNormal distribution with mean of 5.07 and standard deviation of 1.15. **C:** Inter-arrival time distribution for landline calls follows a Weibull distribution with scale and shape of 7200 and 3.2, respectively.

The next component of SSRSM models the calling patterns and spectrum usage. We first describe the session (or calls) generation (SG) module. While calls have been commonly modeled as Poisson processes [53], recent studies on real cellular traffic have suggested different distributions [54], [55]. SG uses a discrete event simulation approach to model calling patterns, using distributions for call duration and arrival rates from those reported in [55]. Figure 5 shows the input statistics we use for generating the mobile and wireline calls.

The event simulator in the SG supports multiple event types for arrivals and departures of mobile and wired calls, extending the model in [12], which only considers wireless calls. Using similar data on arrival rates, as reported in [55] for mobile calls, we model mobile calls arrivals (from cellphones or mobile devices) for every hour and generate arrival events. These events are then processed to create sessions by selecting callees from the caller's social network. The resulting spatio-temporal spectrum usage is measured in terms of the load on the cell towers. The wireline calls are generated using slightly different statistics as depicted in Panel C of Figure 5 and additional constraints such as, access to a wireline device, are placed. For example, individuals moving between activities can use only mobile devices to make calls. Anybody present at a location is assumed to have access to a wireline device in addition to any mobile devices they are assigned. Any individual without a mobile device can make calls only from wireline devices and must be present at a location to be a caller or callee.

The event based simulation provides flexibility to extend SG to include other aspects such as call drops, call retries and busy periods for the individuals. For the experiments in this paper, we use a simpler version in which the callees are chosen uniformly at random from the individual's social network, i.e., no specific relationship between the caller and callee are considered. We also drop calls that terminate at busy individuals (call waiting or call retries are not currently implemented). The individual is considered busy if he/she is already participating in a mobile or a wireline session.

The main metrics we study in this paper are the *Number of Calls* and the *Peak Load* within a time interval, and in some spatial region; the peak load in a cell 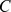 in a time interval 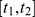 is the maximum number of calls that are simultaneously active in 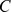 at any time during this interval. We consider both the distributions and average values, as needed. See [12] for more details. Here, for simplicity, we use number of calls as an approximate metric for peak load; and we refer to “number of calls” as load in the rest of the paper.

### Evacuation Model

Let 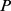 denote the polygon shaped region to be evacuated. Let 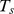 and 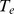 denote the evacuation start and end times, respectively. All the people whose activities take them to the evacuation region 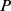 during the interval 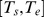 are ordered to evacuate. However only a fraction of them comply, as is often the case in the real world (the remaining stay put in their current location). People who do not enter the evacuation region during this time interval are not evacuated. This results in 3 types of people, who are grouped into 3 classes:

- *Class-1 individuals* are the people whose activities take them into the evacuation region during the time interval 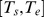, and who comply with the order to evacuate. In our study, we assume that 90% of all people comply with the evacuation order.
- *Class-2 individuals* are people whose activities take them into the evacuation region during the time interval 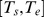, but who do not comply with the order to evacuate. 10% of all the people ordered to evacuate are assumed to be in Class-2. In our study, we choose Class-1 and Class-2 by means of a random 90-10 split of the set of all people whose activities take them into the evacuation region during 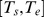.
- *Class-3 individuals* are people who do not enter the evacuation region during the time interval 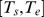, and, therefore, are not ordered to evacuate.

Our study is based on SSRSM, but requires changes and enhancements in the models and implementation of (i) individual activities, (ii) mobility, and (iii) calling pattern. We discuss these changes below.

#### Activities During Evacuation

Individual activities are likely to change in the event of an attack, and are likely to be governed by people's perception of the attack and the steps they take to remain safe. We model the changes in activities to reflect behavioral adaptations of evacuation and compliance, as outlined below. As discussed earlier, all the people whose activities take them to the evacuation region 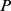 during the interval 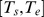 are ordered to evacuate. However, only Class-1 individuals comply and terminate their ongoing activities within the region and immediately evacuate to their home or shelter (in case their home is also in the evacuation region). This is implemented in SSRSM by modifying the activity sequence for such individuals. Class-2 individuals do not comply with the evacuation order, and extend their ongoing activity within the region and remain in the region. Class-3 individuals do not evacuate, but adopt in-place sheltering, and continue the activity they were performing at time 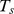 (at the same location). Table 4 presents some basic statistics about the evacuation such as the number of people in each class, total number of cells in the area, cell size, evacuation region etc.

**Table 4:** Some basic statistics of the evacuation.

Item	Information
Number of Class-1 individuals	155,272
Number of Class-2 individuals	17,252
Number of Class-3 individuals	1,428,806
Cells in the evacuation region	12
Total # cells in Portland area	23,124
Cell size	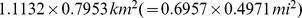
Evacuation region spec:	an octagon^1^ circling the downtown Portland, OR
Road closure (only in Evac I-b and IV)	Burnside Bridge and Morrison Bridge

1 Coordinates of vertices (latitude, longitude): (45.538, −122.665), (45.534, −122.654), (45.527, −122.643), (45.51563, −122.647), (45.507, −122.656), (45.508, −122.687), (45.522, −122.695), (45.532, −122.684).

**Figure 6:**
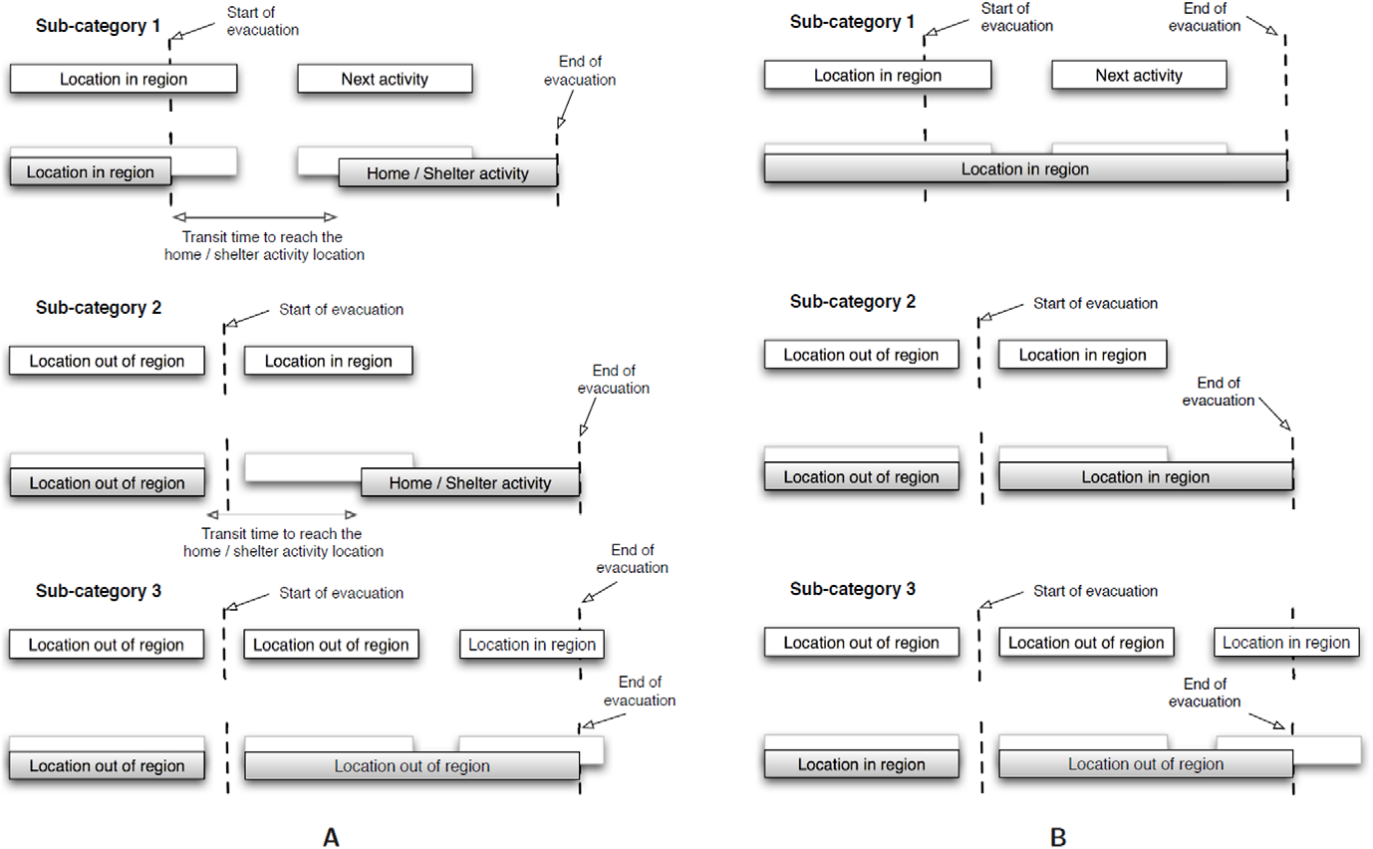
Activity modification for different classes of individuals. **A:** Modifications for Class-1 individuals to incorporate evacuation scenario. Each Sub-category has a slightly different manifestation in the mobility due to evacuation; **B:** Activity modification for Class-2 individuals to incorporate evacuation scenario. Each Sub-category has a slightly different manifestation in the mobility due to evacuation.

**Figure 7:**
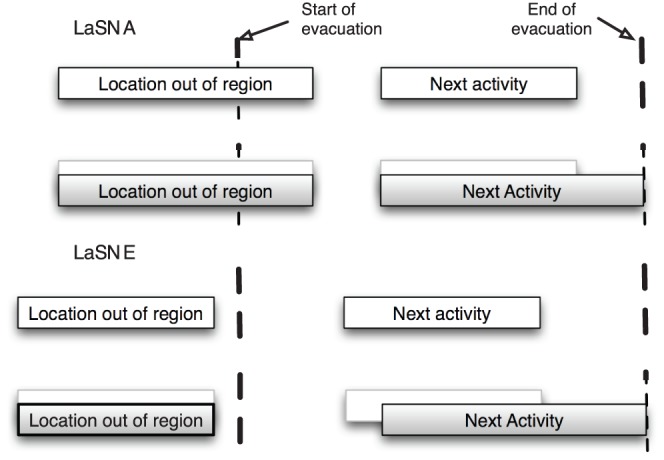
Activity alteration for individuals who are not performing any activity in the region and are completely outside. We consider in-place shelter for some of these individuals due to behavior of individuals.

The above model of activity change is an illustration of the possible responses, and can be altered based on the requirements. A detailed model of updated activities for evacuation has been created. Class-1 and Class-2 individuals are further divided into 3 sub-classes based on whether they are inside the evacuation region, in transit, or outside the evacuation region at the beginning of evacuation. All Class-3 individuals behave the same way since they are outside the evacuation region and plan to stay out. The details on updated activities and the sub-classes are available in Appendix S1. These details are also displayed schematically in Figures 6 and 7.

#### Mobility and Routing

When people evacuate, they have to choose routes to get to their destination (either their home or the shelter), and this is likely to cause congestion in the roads out of the evacuation region. Further, in two of the scenarios, we assume two of the bridges in the area are closed, so that people have to choose alternative routes. Choosing shortest paths for all people would cause very high congestion, and in order to incorporate the realism of road traffic, we use a loading algorithm to choose evacuation routes for people. The route selection module involves the following components.

- a *road network representation* using data from the NAVTEQ Geographic database for the transportation system. Any changes to infrastructure, e.g. road closure, need to be added to this representation.
- a *road network updater* to reflect the effect of changes in traffic patterns along the time axis on the road links.
- a *route constructor* that finds permissible short routes for trip requests (source-destination pairs) with the updated road network. These routes are subject to traffic rules, and are also constrained by permissible travel modes, capacity and potential damage to parts of the infrastructure.

The road network updater [56], [57] captures the impact of road conditions on traffic throughput. The average traveling speeds (which reflects throughputs) on the links are initialized to maximum allowable values. Intuitively, as the simulation proceeds, the traffic loads on the links gradually increase. The time-varying loads on links, representing the “congestion”, in turn feed back to the average traveling speeds of links, such that the average speed of a link is lowered when the link gets more congested, and vice versa. Thus, the road network updater uses iterative network loading to periodically monitor the changes in traffic patterns and updates the weights (as a function of loads) on the roads segments. This is done at a preset frequency, so that the route constructor dynamically produces shortest routes for each trip (i.e., a source-destination pair) with respect to the latest road condition (i.e., average travel speed, traffic load). Trip times are determined dynamically by road conditions along the links traveled. The mobility so computed is converted into dwell times within each cell tower area. In the evacuation cases, people will stay at the activity location following their first trip (e.g., to the shelter or home) after the evacuation order is made.

### Changes in Calling Pattern and Scenarios

The SG module is discussed in Section *Modeling calls and usage patterns*. Here, we outline the changes made to the calling patterns to capture the behavioral changes during an evacuation. In such a scenario, it is normal for individuals to notify their family and friends about their current location and condition, leading to an increase in the volume of calls in the evacuation region and outside. We have modified SG to model the increased load with respect to both wireline and wireless calls. In case of wireline, the individuals make calls before the actual evacuation begins. This can be attributed to individuals who hear rumors about the impending evacuation or notice pollution or smell in the air. We consider a time duration of 30 minutes before the evacuation begins as the target interval and create a random number of additional wireline calls with only actual evacuees as callers (Class-1 individuals). Mobile calls are generated all through the evacuation duration from 12 Noon till 3:00PM and are made with varying scales to study the effect of the calling patterns on the load at each cell tower.

We consider a parameterized study where different parameters are considered for the wireline calls, wireless calls and duration of the calls for the different classes of people. For each set of the parameters we also study the effect of multiple road closures. Specifically, we consider five scenarios; a base case and 4 evacuation scenarios. In each of the evacuation scenarios the wireless and wireline call rates go up by a factor but the duration of the calls drop. For simplicity, we assume that the wireline and wireless calls have the same duration. All the scenarios and their associated parameters used in our simulation are as shown in Table 5. The *Base Case* corresponds to the calling pattern with no evacuation; where all individuals engage in their normal daily activities and sessions (including wireline and wireless calls) according to the original models. In base case, the wireless rate, the wireline rate and the call duration (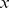, 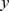 and 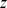) are as described in Figure 5 and are based on parameters taken from [55]. The session durations follow a LogNormal distribution with mean of 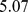 and standard deviation of 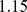. The wireline call rate follows a Weibull distribution with scale and shape of 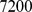 and 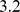, respectively.

**Table 5:** Simulation study cases.

Case	Individual Class	Wireless Rate	Wireline Rate	Call Duration	Road Closure
Base	Class-1	x	y	z	No
	Class-2	x	y	z	
	Class-3	x	y	z	
Evac I-a	Class-1	5x	10y	0.5z	No
	Class-2	3x	5y	0.5z	
	Class-3	2x	2y	0.5z	
Evac II-a	Class-1	10x	10y	0.75z	No
	Class-2	5x	5y	0.75z	
	Class-3	2x	2y	0.75z	
Evac I-b	Class-1	5x	10y	0.5z	Yes
	Class-2	3x	5y	0.5z	
	Class-3	2x	2y	0.5z	
Evac II-b	Class-1	10x	10y	0.75z	Yes
	Class-2	5x	5y	0.75z	
	Class-3	2x	2y	0.75z	

**Figure 8:**
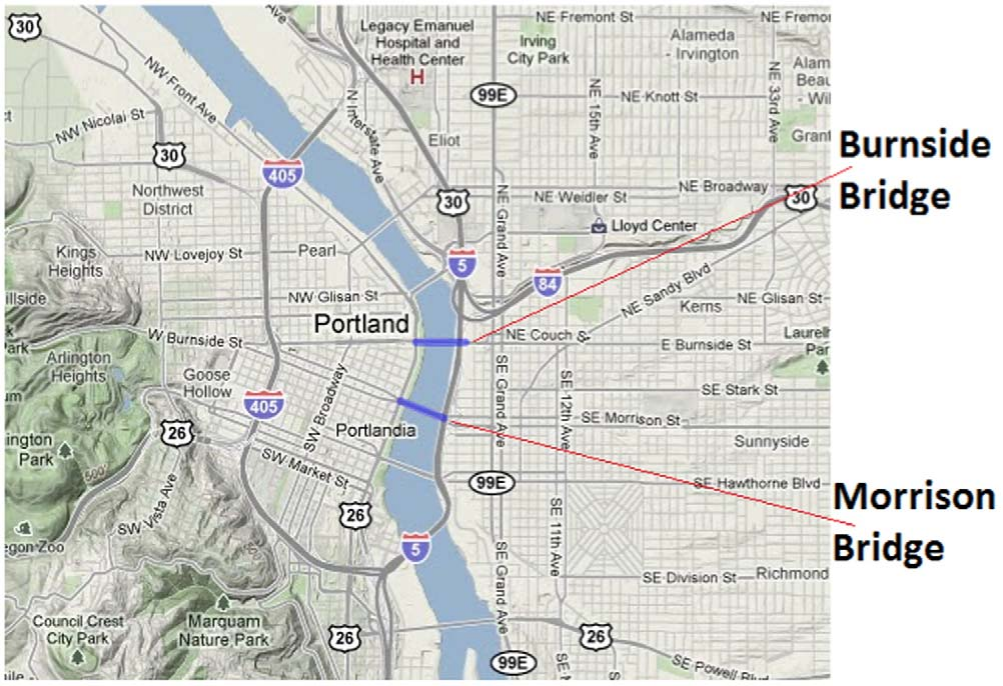
Locations of Burnside Bridge and Morrison Bridge which are closed in Evacuation Scenarios Evac I-a-b and Evac II-a-b.

*Evac I-a* and *Evac II-a* describe the evacuation scenarios in which people's activities and mobility is altered (as discussed in the subsection *Activities during evacuation* of Section *Formal Models and Methodology*) and the call rates and durations change. The difference between *Evac I-a* and *Evac II-a* is that they have different set of parameters for the rate and duration of the wireless and wireline calls. *Evac I-b* and *Evac II-b* are the corresponding variants of Evac I-a and Evac II-a, with road closures on Burnside Bridge and Morrison Bridge. Figure 8 shows the locations of these two bridges.

Under each of the scenarios, we perform a parametric evaluation of the altered calling patterns on the overall load of the base stations. For every scenario outlined above, we vary the proportions of calls originating from the 3 classes of individuals and observe the effect on the load of the base stations. This will capture the effect of different behavior patterns on the cell tower loads.

### Hybrid Cellular and Multi-Hop Mesh/Ad-Hoc Networks for Increasing the Capacity

In situations where fixed cellular infrastructures (base stations) is almost fully loaded, integrating it with multi-hop mesh networks has been proposed as a way of increasing the capacity; overloaded cells could then move some of the excess calls through the multi-hop network to other less loaded base stations or other gateways. This is particularly useful in settings such as evacuation, where such a mesh network could be set up rapidly. However, the additional hops could lead to delays and adversely effect the quality of service, leading to trade-offs between the delays, throughput and total power usage. We formalize this as the MaxHybridNetworkCapacity (MHNC) problem below. Let 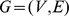 denote a hybrid network with 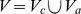, where 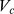 and 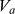 denote the cellular and mesh nodes, respectively. 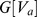 denotes the multi-hop network constructed by the mesh nodes. Let 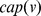 denote the capacity of each 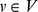. Given a load vector 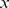 and a delay parameter 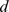, where 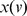 denotes load on node 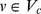, and 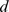 denotes the maximum number of hops allowed on the multi-hop network, the goal of the MHNC problem is to construct a function 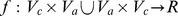, so that (i) 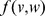 denotes the load from node 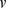 to node 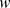, 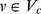 that is routed to 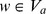 (through the multi-hop network), (ii) 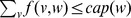, (iii) for each 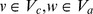, 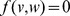 if 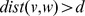, and (iv) the total load that is serviced is maximized.

The MHNC problem is NP-complete in general (by a reduction from a variant of the Dominating Set problem), and in this paper we study a simple iterative load assignment approach, GreedyLoadBal, for this problem. For simplicity, we assume that nodes in 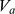 are co-located with the base stations, and the mesh network only routes calls from overloaded base stations to other base stations with spare capacity. We consider a simple iterative load assignment approach: in each iteration, each base station 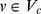 transfers its excess load 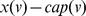 to all its neighbors 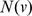 equally; this is repeated 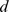 times, so that the load is not moved more than 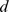 hops. When the above algorithm stops, many cell towers might still have load 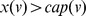, and in such cases, the excess calls would be dropped. We observe that the load does get dispersed on a larger region, and the fraction of dropped calls reduces significantly.

**Figure 9:**
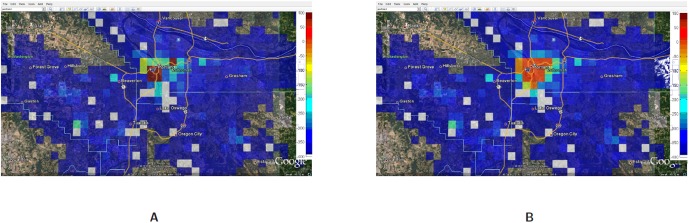
Evac I-a: Deficit capacity at each individual cell before and after the cascading between the time interval 1:00PM–1:30PM. **A:** Deficit capacity at each cell without cascading. A negative deficit means lack of capacity to process calls. The capacity is estimated as a scaled value of the normal load (scale = 2, in this case); **B:** Deficit capacity at each cell after cascading. The cells that have excess load shed their calls to neighboring cells for up to 10 hops with neighbors chosen at random.

**Figure 10:**
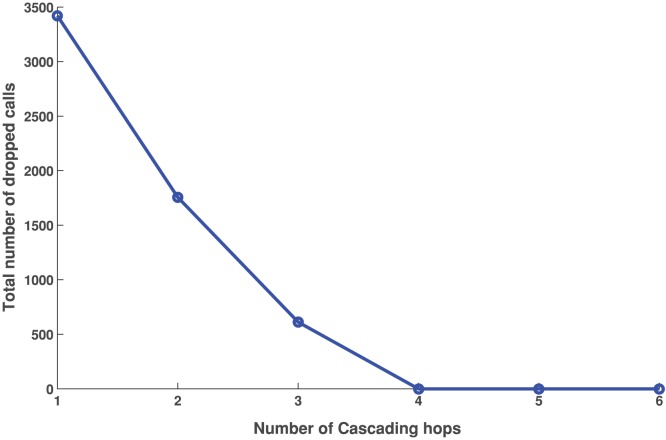
Number of calls drops when the cell towers are allowed to shed excess load to the neighboring base stations which are a number of hops away. The number of dropped calls reduce as the number of hops increase. The scenario corresponds to noon time during evacuation with roads closed, i.e. Evac II-b. The number of calls dropped reduce to 0 beyond 4 hops.

Figure 9 shows the load in each cell with and without running GreedyLoadBal: Figure 9A shows the excess or deficit (i.e., the difference between the capacity and the total load) in each cell as a result of the evacuation, without any hybrid network for balancing the load. We assume that the capacity is 50% more than the load under normal condition, which reflects the normal provisioning that service providers allow. This scale is a parameter which can be easily altered in our simulations but note that the results are sensitive to this parameter. The positive values on the scale in Figure 9A indicate a deficit in the cell and negative values indicate a surplus. All the cells that have a zero or no residual capacity are marked by dark red colored cells. Figure 9B shows the capacity deficit at each cell after running GreedyLoadBal, with a distance parameter of 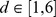, i.e., the calls can potentially take up to 1 through 6 hops to be handled in case capacity is not available. Observe that hybrid network spreads the load on almost twice as many base stations, as a result of the load balancing. The red and green squares in Figure 9A are being replaced by orange squares in Figure 9B and some of the blue squares are changed to green. Lot more cells become orange as they change either from being a surplus cell to a near-zero cell, or from a deficit cell to a near-zero cell when load is added or removed respectively due to load-balancing. Figure 10 shows that as the number of hops allowed for the excess load to be shed increases from 1 to 2, the total number of dropped calls reduces by a half, i.e., from 3500 to 1750. With 3 hops allowed the total number of dropped calls reduces to almost 500, showing a non-linear improvement in successful call sessions.

## Results and Analysis

This section describes the effect of different levels of compliance by evacuees, on the congestion in the communication and transportation networks. A summary of main findings is given at the end.

### Sensitivity Analysis of Evacuation Compliance

This section examines the sensitivity of the compliance rate to the base station loads, spatial distribution of people and their mobility. We consider 3 different compliance rates i.e. 90%, 80% and 70%. Compliance rate is the percentage of population in the evacuation region that obeys the evacuation orders. As explained earlier under Section *Evacuation Model*, who comply with the evacuation order become the Class-1 individuals and the non-compliant population (10%, 20% and 30% respectively) becomes the Class-2 individuals.

**Figure 11:**
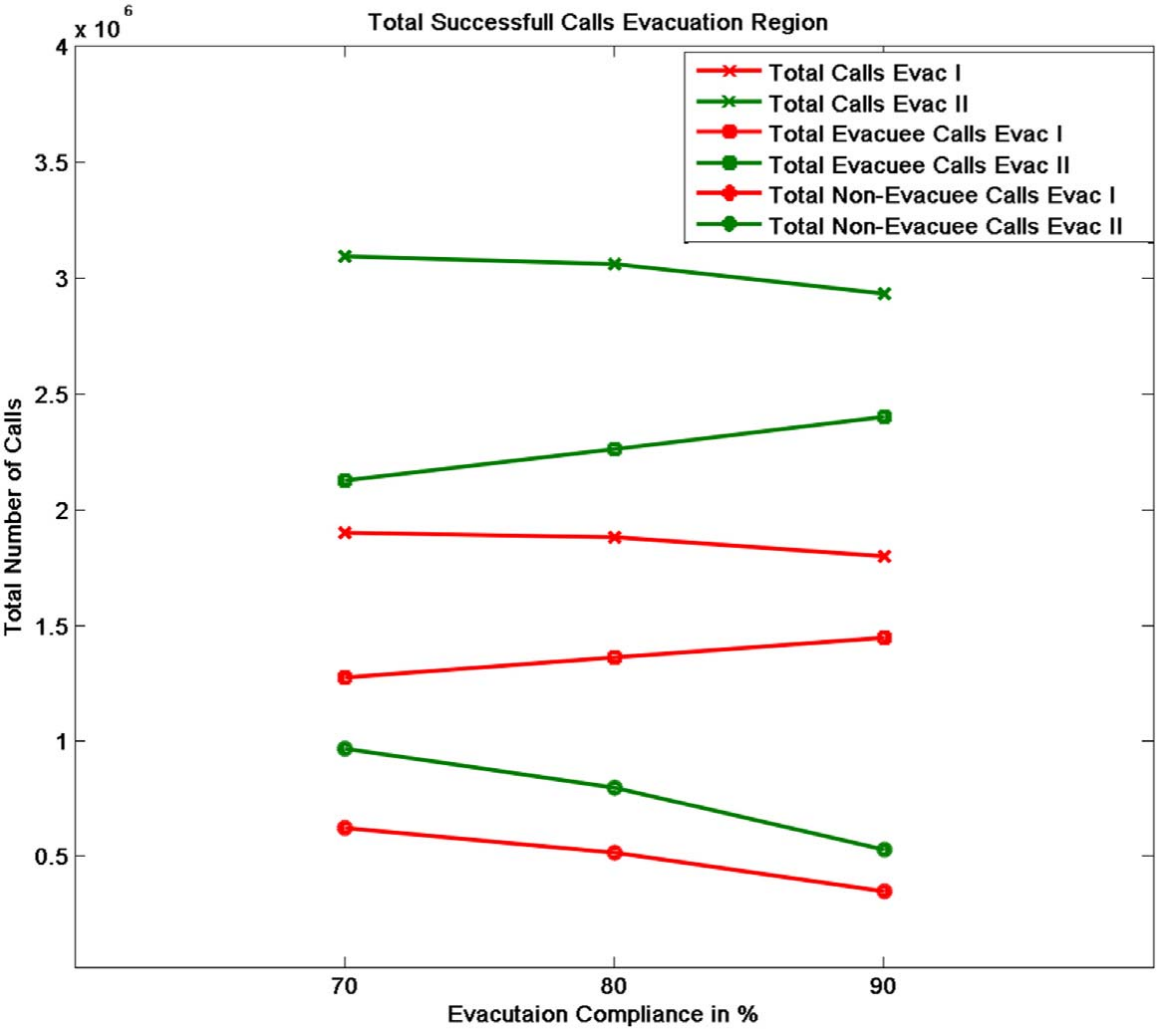
Number of successful calls in evacuation region made by the evacuees and non-evacuees for different compliance rates.

**Figure 12:**
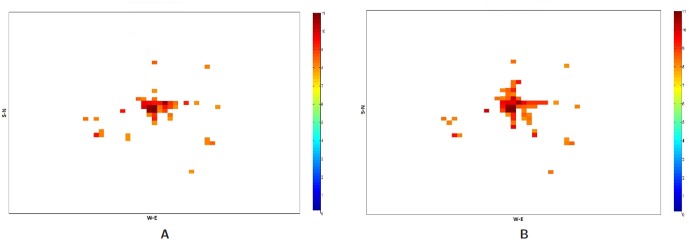
Overloaded base stations with threshold set at 250 calls: Spatial and temporal view of load in Portland region between the time interval 12:00PM–12:30PM for the Evac cases I-a and I-b with 90% Compliance. The numbers are in 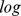 scale. **A:** Load in the Portland region during Evac I-a scenario with activities and calling patterns corresponding to an evacuation with no road closure with 90% evacuation compliance; **B:** Load in the Portland region during Evac I-b scenario with activities and calling patterns corresponding to an Evacuation with road closure with 90% evacuation compliance.

**Figure 13:**
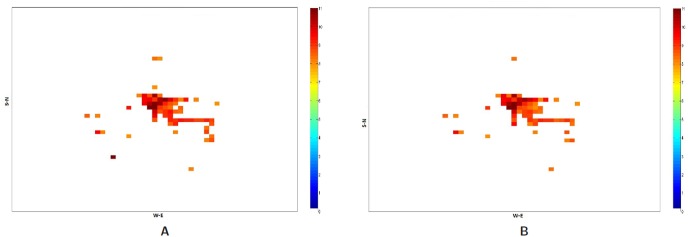
Overloaded base stations with threshold set at 250 calls: Spatial and temporal view of load in Portland region between the time interval 12:00PM–12:30PM for the Evac cases I-a and I-b with 70% compliance. The numbers are in 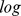 scale. **A:** Load in the Portland region during Evac I-a scenario with activities and calling patterns corresponding to an Evacuation with no road closure with 70% evacuation compliance; **B:** Load in the Portland region during Evac I-b scenario with activities and calling patterns corresponding to an Evacuation with road closure with 70% evacuation compliance.

**Figure 14:**
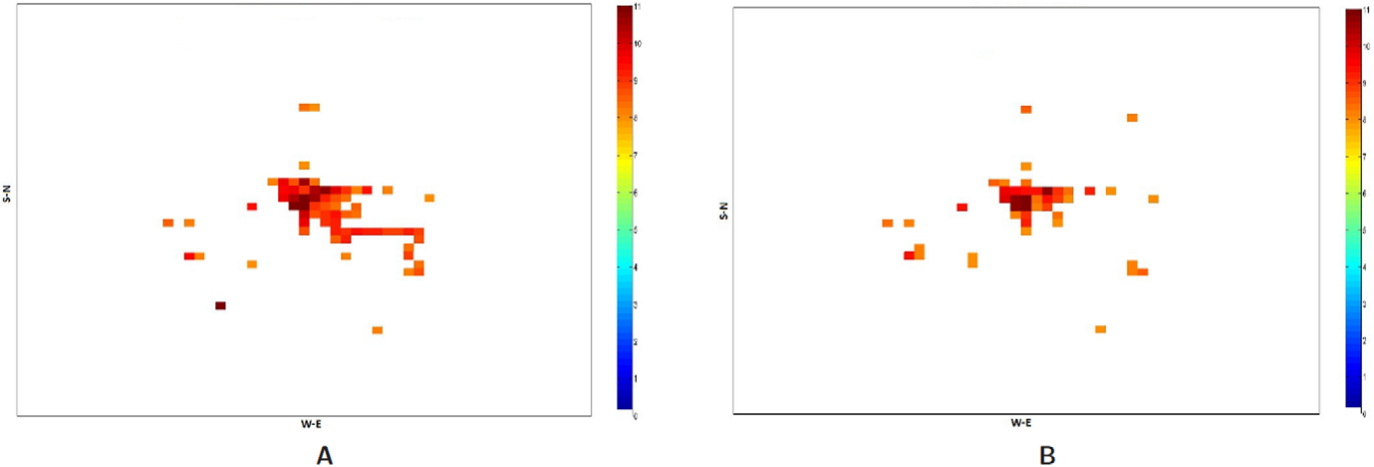
Overloaded base stations with threshold set at 250 calls: Spatial and temporal view of load in Portland region between the time interval 12:00PM–12:30PM for the Evac cases I-a 70% and I-a with 90% Compliance. The numbers are in 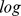 scale. **A:** Load in the Portland region during Evac I-a 70% compliance scenario with activities and calling patterns corresponding to an Evacuation with no road closure; **B:** Load in the Portland region during Evac I-a 90% compliance scenario with activities and calling patterns corresponding to an evacuation with no road closure.

**Figure 15:**
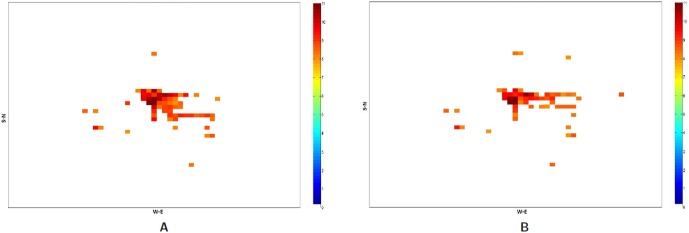
Overloaded base stations with threshold set at 250 calls: Spatial and temporal view of load in Portland region between the time interval 12:00PM–12:30PM for the Evac cases I-b 70% and I-b with 80% Compliance. The numbers are in 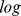 scale. **A:** Load in the Portland region during Evac I-b 70% compliance scenario with activities and calling patterns corresponding to an evacuation with road closure; **B:** Load in the Portland region during Evac I-b 80% compliance scenario with activities and calling patterns corresponding to an evacuation with road closure.

The relative increase in the call frequency is higher for the evacuees as compared to the non-evacuees although both classes of people (class I and II) increase their call rate relative to the base rate. This means that as the compliance rate increases, the number of evacuees increase and hence the overall call volume should increase. Figure 11 plots the total number of successful calls, calls by evacuees and calls by non-evacuees. Contrary to our expectation, the figure shows that as the compliance rate increases from 70% to 90%, the total number of successful calls marginally drop. This is even after the fact that there are more evacuees and they are making calls at a higher rate than the non-evacuees. This phenomenon holds under both evacuation scenarios I and II. A careful analysis shows that higher call volume caused by more evacuees leads to concentrated loads on some base stations since higher traffic congestion confines them to a smaller geographical area. Table 6 and Table 7 show the number of base stations that meet or exceed the capacity threshold defined for a congestion level. Table 6 assumes that a base station can hold only 250 calls simultaneously beyond which it gets overloaded and Table 7 assumes that it can hold 750 simultaneous calls. Using these indicators of congestion for a base station, we analyze the impact of higher number of evacuees on the spatial distribution of people through their mobility and its effect on the communication network usage. Some non-intuitive and emergent phenomenon observed from the analysis of the communication load immediately after the evacuation start time [12.00–12.30PM] are as follows shown in Figures 12, 13, 14 and 15:

1. At a lower compliance rate, because of fewer evacuees, the spatial distribution of people is more even as compared to the distribution at the higher compliance rate. As people are more spread out, the base station load becomes more distributed. Figure 14 shows the congested base stations (assuming 250 calls as the congestion threshold) for compliance rate 70% and 90%. Surprisingly, at compliance rate of 70%, there are many more congested base stations than at 90%. This is because at 90%, as people are trying to flee the evacuation region and fight the traffic congestion, their locations become spatially concentrated which makes the base station load also more concentrated to a few base stations.
2. Figures 12 and 13 show that at 90% compliance, road closures increase the base station congestion but at 70%, the effect is very marginal. Three are only a few more base stations that get overloaded due to road closures. This is possible because once the main bridges have been closed, people find different short cuts to get to their destination.
3. Figures 14 and 15 show that at higher compliance, “no road closure” scenario has fewer base stations congested than the “road closure” scenario where people mobility scatters across many cells as the main bridge is closed. In our mobility routing, a shortest path is assigned for each individual during evacuation which leads to higher congestion on the bridge as it provides a major connection to the destination shelter. When the bridge is closed, people are routed via other roads and the load is distributed more evenly across the base stations.

**Table 6:** Congestion analysis for time 12:00PM–12:30PM with call capacity threshold = 250 simultaneous calls.

Case	Evacuation Compliance	Congested Cell Towers ( 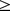 Threshold)	Road Closure
Evac I-a	90	46	No
Evac I-b	90	60	Yes
Evac I-a	80	42	No
Evac I-b	80	58	Yes
Evac I-a	70	68	No
Evac I-b	70	65	Yes

**Table 7:** Congestion analysis for time 12:00PM–12:30PM with call capacity threshold = 750 simultaneous calls.

Case	Evacuation Compliance	Congested Cell Towers ( 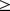 Threshold)	Road Closure
Evac I-a	90	7	No
Evac I-b	90	12	Yes
Evac I-a	80	12	No
Evac I-b	80	10	Yes
Evac I-a	70	16	No
Evac I-b	70	14	Yes

### Analysis of People Mobility

**Figure 16:**
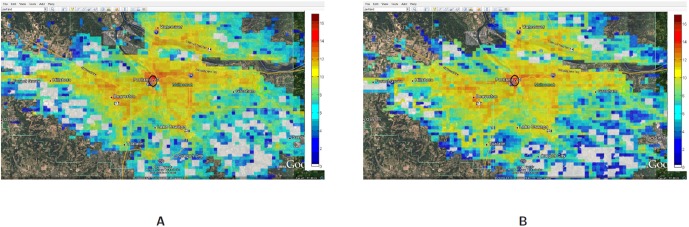
People Intensity in base case and evacuation case with no road closure, Evac I-a. Numbers represented in 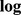
 **scale.** **A:** People Intensity in base-case signifying the number of people in each cell block in the city during normal day; **B:** People Intensity in Evac I-a signifying the number of people in each cell block in the city during evacuation with no Road Closure.

We use *People Intensity* (PI) and *Mobility Intensity* (MI) to measure the presence and movement of people across cells. PI(c,t) is measured for cell 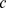 and time interval 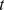, as the number of distinct people that have been in 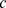 during 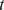; and PI(c) = 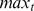 PI(c, t) is the maximum number of people in cell 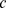 among all the time intervals. Note that people who travel across multiple cells during 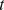 are counted in each of those cells. Mobility Intensity for time interval 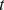, MI(t) is measured as the average number of cells traveled per person during 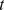. For both PI and MI, 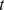 is set at half an hour. Figure 16 shows a heat map of PI for base case vs. Evac I-a scenario. The people intensity goes up in the evacuation case compared to the base case as they get stranded on the roads for longer time during the evacuation.

**Figure 17:**
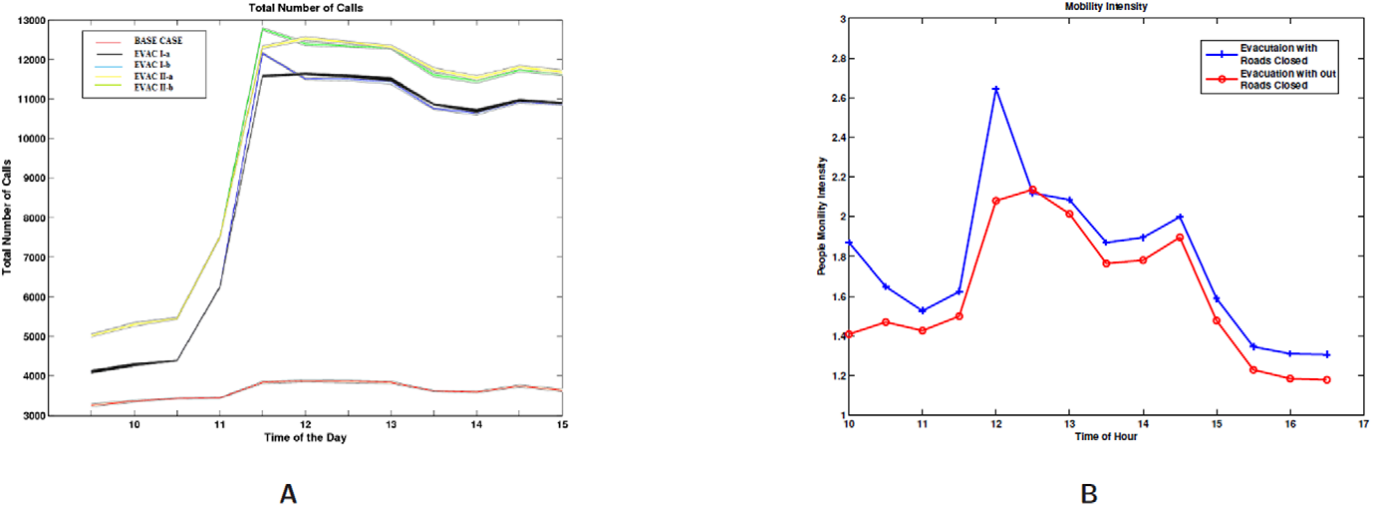
Total number of calls and mobility intensity in different scenarios. **A:** Total number of calls made under each scenario for the entire region of Portland, before, during and after evacuation hours, shaded region indicates the 95% *Confidence Interval* over 10 Iterations; **B:** Mobility intensity signifying average number of cells traveled by a person in each hour. Mobility Intensity under evacuation case with and without road closure.

Figure 17A shows the total number of calls under each of the scenarios for the entire region of Portland. The total number of calls go up during evacuation compared to the base case. Evac II-a has higher number of calls starting at 11am compared to Evac I-a and the base case. Similarly the number of total calls go up Evac I-b and Evac II-b representing road closure effect as compared to Evac I-a and Evac II-a.

Figure 17B shows the mobility intensity under evacuation with and without road closure. The mobility intensity does not change under the two evacuation scenarios where the call volume is different. It only changes from the base case to evacuation case and from road-closure to no road-closure. Mobility Intensity under Evac II-a vs. Evac II-b scenarios shows that the congestion caused by the road closure increase the mobility intensity for all hours. This is because the roads are closed for the entire day and not just during evacuation hours. During evacuation hours this difference is amplified. The road closure scenarios have been studied to see how small disruptions in infrastructures can amplify a crisis.

### Main Observations

Our main observations are the following.

**Figure 18:**
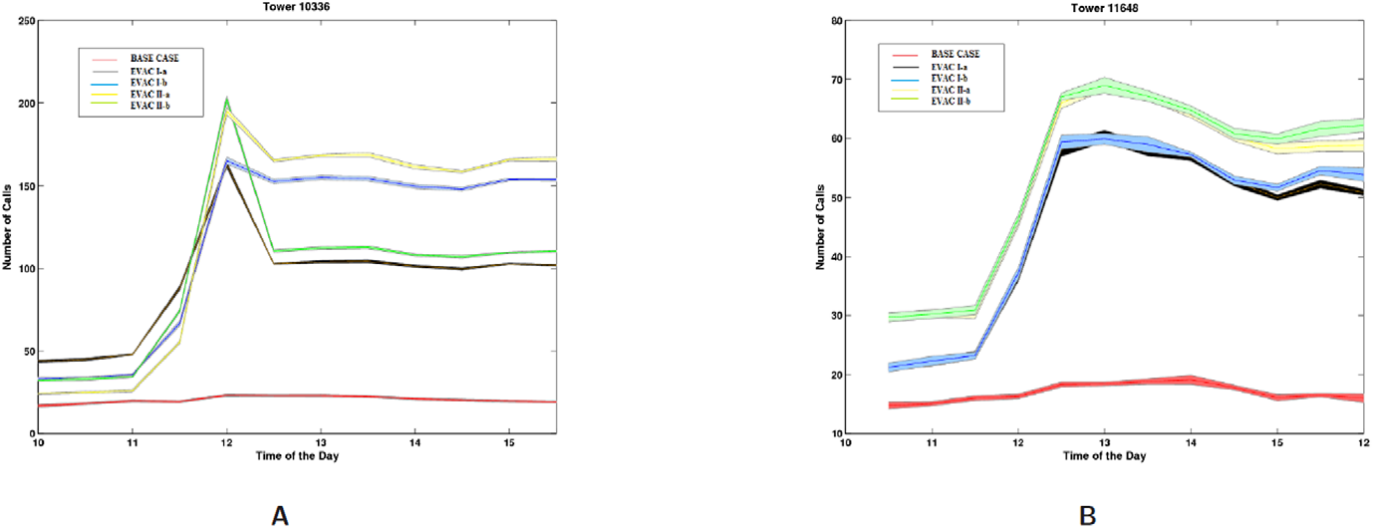
Number of Calls during each evacuation scenario at cell towers corresponding to different regions, shaded region indicates the 95% *Confidence Interval* over 10 Iterations. **A:** A cell belonging to the evacuation region. The entire cell may not be inside the region; **B:** The cell consisting the shelter area to which the people are evacuated.

**Figure 19:**
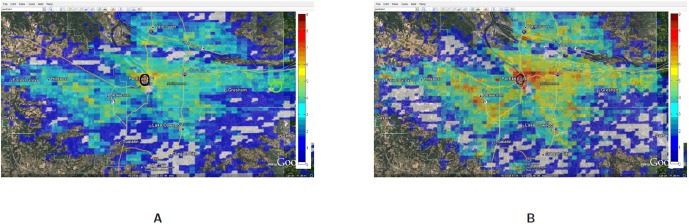
Spatial and temporal view of the load in the Portland region between the time interval 1:00PM–1:30PM for the base case and evacuation case Evac I-a. The numbers are represented in 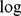 scale. **A:** Load in Portland during a normal day with activities and calling patterns corresponding to base case; **B:** Load in Portland during an evacuation case Evac I-a.

**Figure 20:**
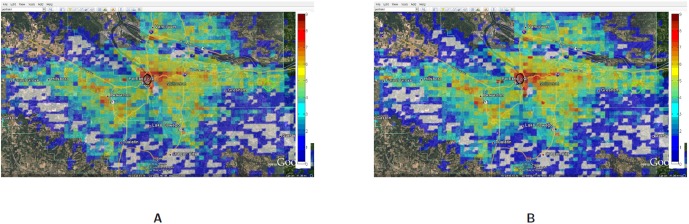
Spatial and temporal view of load in Portland region between the time interval 1:00PM–1:30PM for the Evac cases I-a and I-b. The numbers are in 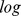 scale. **A:** Load in the Portland region during Evac I-a scenario with activities and calling patterns corresponding to an Evacuation with no road closure; **B:** Load in the Portland region during Evac I-b scenario with activities and calling patterns corresponding to an Evacuation with road closure.

**Figure 21:**
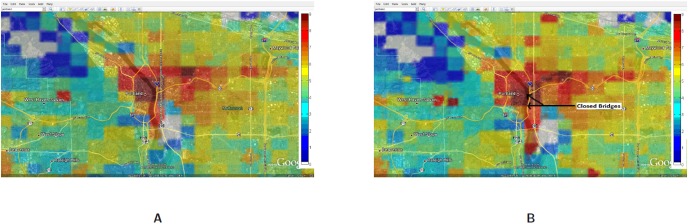
Spatial and temporal view of load in Portland region between the time interval 1:00PM–1:30PM for the Evac cases I-a and I-b zoomed in to Downtown region. The numbers are in 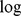 scale. **A:** Load in the Portland region during Evac I-a scenario with activities and calling patterns corresponding to an Evacuation with no road closure; **B:** Load in the Portland region during Evac I-b scenario with activities and calling patterns corresponding to an Evacuation with road closure.

**Figure 22:**
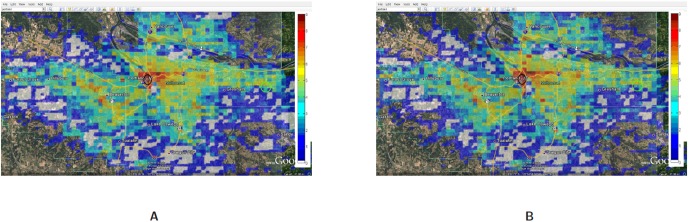
Spatial and temporal view of load in Portland region between the time interval 1:00PM–1:30PM for the Evac case II-a and II-b. The numbers are in 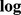
 **scale.** **A:** Load in the Portland region during Evac II-a scenario with activities and calling patterns corresponding to evacuation with no road closure; **B:** Load in the Portland region during Evac II-b scenario with activities and calling patterns corresponding to evacuation with road closure.

1. Figure 18 shows how the load temporally changes in a typical evacuation cell and a non-evacuation cell during the evacuation time. Figure 18A shows the load in a cell that belongs to the evacuation region. Figure 18B shows the load in one of the non-evacuation cells that is being used en route to the shelter. As people leave the evacuation location the mobile call volume increases from 40 calls (base case) to over 300 calls (Evac II-b) in this cell. In the non-evacuation cell, Figure 18B, the call volume changes from almost 0 to 65–70.
2. Even in the base case the load is higher in the downtown Portland area as compared to the overall region as shown in Figure 19. This is because the calls are distributed based on the population density of the region, and the downtown area has more people in the day time.
3. Figure 19 compares the spatial and temporal view of the load during evacuation between 1:00PM to 1:30PM with the load on a normal day (or base case). This zoomed in view shows that the load (number of simultaneous calls) has increased 3–4 folds in and around the downtown region. As individuals try to move out of the downtown evacuation region, marked by a black circle, the call volume increases in the evacuation area as well as the shelter area (marked by a green circle and located directly to the east of the downtown area).
4. A zoomed in view of the comparison of Evac I-a scenario with Evac I-b in Figure 20 and Figure 21 highlights the difference in load arising due to road closure. Figure 21B points the two bridges that are closed and the extra load in the area caused by this closure. A clearer view of the closed roads can be seen in Figure 8. However, Figure 22 shows that the increase in the load in Evac II-a is not as high as that in Evac I-a in Figure 20. This is because the call rate increases several folds in Evac II-a as compared to Evac I-a, which increases the number of calls. The increased number of calls result in more and more callees being busy which increases the number of call drops.
5. Figure 17 compares the mobility intensity with and without road closure from 10:00AM to 4:00PM. The road closure causes people to take alternative routes to their destination. These are not likely to be the shortest paths causing them to travel through more cells and hence increasing the MI. Note that the roads are closed for the entire day and hence the MI is higher even during non-evacuation hours.
6. The cell towers are equipped to shed load to neighboring base stations when overloaded. We study the effect of offloading excess calls to base stations several hops away. A parameterized study is performed on the number of hops a call is allowed to make. Experimental results in Figure 10 show that it does not pay to increase the number of cascading hops beyond 4. At 4 hops the number of dropped calls reduce to zero.

### Summary and Conclusions

The paper highlights the role of human initiated inter-dependencies between critical societal infrastructures. A case study is used to demonstrate how a chemical plume in the downtown Portland, Oregon area causes evacuation of the individuals which further causes vehicular traffic congestion. This impacts the geographical distribution of the people and when combined with a modified cell phone usage pattern, it results in overloading of the base stations in non-intuitive ways. A multi-hop network is utilized to augment the capacity so that the base stations can shed load to the neighboring base stations several hops away.

A modeling environment that is individual based, and combines a number of public and commercial data sets with statistical and dynamic models is used to represent the idealized scenario. A multi factorial experimental design and sensitivity analysis show non-linear effects of changes in the compliance level of evacuees. For example an evacuation compliance rate of 70% results in higher number of overloaded base stations than the evacuation compliance rate of 90%. This paper provides a systematic analysis of the cascading failures in multiple societal infrastructures and highlights the technical challenges encountered in a crisis as a result of the interactions among large infrastructural systems.

## Supporting Information

Appendix S1 **List of assumptions and detailed activity model for evacuation.**
(PDF)
