# Supplementary material for: Human Initiated Cascading Failures in Societal Infrastructures
Source: PLoS One. 2012 Oct 31;7(10):e45406. doi: 10.1371/journal.pone.0045406 (PMC3485300; doi:10.1371/journal.pone.0045406)
Supplement: Appendix S1 — List of assumptions and detailed activity model for evacuation. (PDF) [file pone.0045406.s001.pdf]

APPENDIX S1

List of Assumptions

We list below all the assumptions in our current study and implementation.

Scenario

- The plume is abstracted out as a one-time event in a polygonal region. We do not take the physical and dynamical aspects of the plume into account.

- The logistics of propagating information about the evacuation orders, and implementing them are not explicitly taken into account; the "compliance rate" among the evacuees partially takes this into account. Individuals outside the evacuation region are assumed to comply fully with the "in-place shelter" orders.

Transportation Network

- All people being routed are assumed to travel by car, and do not switch cars during the trip.

- The road traffic density is not completely calibrated by explicit traffic measurements, and no micro-simulation is performed to update the routes and location choice, based on traffic congestion. The base traffic and spectrum demand is computed using shortest path routes for each individual, which do not take road capacities and speed limits into account.

- The routes chosen by people who evacuate, and the resulting traffic density are computed by a loading algorithm which updates the loads for each road segment periodically.

Communication Network

- The entire region is partitioned into uniformly sized and square-shaped cells.

- There can be one and only one cell tower in each cell.

- Each cell tower accounts for all service providers inside the same cell area.

Social Network

- The social network considered here reflects the people with whom the person interacts based on the sub-location modeling at activity locations [1].

Devices C Phones

- The mobile devices are allocated based on the demographics of the population [2]. As a result, a single person can have multiple devices. In such cases, one device is selected at random to participate in the session.

Call Sessions

- Load is assumed to be the number of simultaneous sessions that happens on a given base station inside a given time interval.

- Time scale in session generation is one second.

- Even though we generate data sessions, we do not use specific statistics for data sessions to generate the inter-arrival and duration times. We just mark some sessions randomly as data sessions.
- Callers are selected at random but the callees are picked based on callers' social networks.
- Currently, the duration of the call does not depend upon the type of the social contact between the caller and the callee.
- We do not consider features such as call-waiting and retries while generating sessions. These can be easily added into the session simulator with more events to handle such conditions. In the current implementation, the individuals are marked as *busy* if they are already participating in a session. The busy individuals cannot be picked as a callee. The calls are dropped when we do not find an available callee from the social network of the caller.

Detailed activity model for evacuation

We now provide further details on the process of altering the altered activities and changed mobility resulting from the evacuation. The entire population is divided into 3 classes of people. Class-1 and 2 are either in the evacuation region or have activities scheduled in the evacuation region so they need to be evacuated. Class-3 people are outside the evacuation region and have no activities scheduled in the evacuation region so they do not need to be evacuated. They continue to do the same activity they were doing at Noon, unless they are in transit, in which case they go home, or to the shelter. The Class-1 and 2 people are further sub-divided into 3 categories based on where they are at Noon. Each sub-category is handled differently during the simulation as shown in Figure 6.

(i) *Sub-category 1* (SC1) are the individuals who are at one of the locations inside the evacuation region at Noon (or the start of the evacuation) and their next activity locations happens to be in the evacuation region as well.

(ii) *Sub-category 2* (SC2) are the people who are in transit inside the evacuation region.

(iii) *Sub-category 3* (SC3) are individuals who are currently outside the evacuation region but have activities scheduled to be performed inside the evacuation region during the evacuation period.

Class-1 individuals

Among Class-1, the individuals belonging to SC1 terminate their current activity at the start of the evacuation and proceed to evacuate to home or shelter. They go to the shelter if the home is located inside the evacuation region.

For SC2 individuals, the activity following the start of the evacuation is replaced to a home or shelter activity. In other words, people in transit are being sent home or to a shelter. Shelter (or home) activity is selected (as before) if home is located inside (or outside) the evacuation region, respectively.

SC3 individuals remain at the location of the activity that was being performed at noon (or the beginning of the evacuation). Since this location is outside the evacuation region, it works as a "in-place" shelter.

Class-2 individuals

Class-2 individuals are the ones who are not in compliance with the evacuation orders. Unlike Class-1, Class-2 people remain in the region even when the evacuation order is in effect. Among Class-2, the individuals belonging to SC1 continue to perform their current activity and remain at that location till the end of the evacuation. They do not leave for home or shelter when evacuation order is given.

Among the SC2 individuals, the activity immediately following the start of the evacuation that is in a location inside the evacuation region is retained and extended till the end of the evacuation time. The SC2 individuals do not leave this location till the end of the evacuation.

For SC3 individuals, the activity scheduled at the start of the evacuation is performed as usual, but the individual remains at this location till the end of the evacuation. The duration of the activity is extended and the individual remains at this location for the duration of the evacuation.

Class-3 individuals

The sub-categories outlined above do not apply to the Class-3 individuals. These individuals are outside the evacuation region and have no activities planned in the evacuation region. These individuals use either their current location as "in-place" shelter or go home or to the shelter. Figure 7 shows that everyone's activities' in this class are modified in the same manner.

References

1. Bisset K, Marathe M (2009) A cyber-environment to support pandemic planning and response. DOE SciDAC Magazine : 36–47.

2. Beckman R, Channakeshava K, Huang F, Kumar VSA, Marathe A, et al. (2010) In: Synthesis and Analysis of Spatio-Temporal Spectrum Demand Patterns: A First Principles Approach. pp. 1 -12.
